# NP Role and Patient Outcomes Are Supported by High-Level Evidence. Comment on Almutairi et al. Nurse Practitioner: Is It Time to Have a Role in Saudi Arabia? *Nurs. Rep.* 2020, *10*, 41–47

**DOI:** 10.3390/nursrep12020040

**Published:** 2022-06-09

**Authors:** Julee B. Waldrop

**Affiliations:** School of Nursing, Duke University, 307 Trent Drive, Durham, NC 27710, USA; julee.waldrop@duke.edu

**Keywords:** nurse practitioner, nurse practitioner role, nurse practitioner practice, patient outcomes

## Abstract

This is a letter to the editor in response to the article titled “Nurse Practitioner: Is it Time to Have a Role in Saudi Arabia?” Clarifications on the nurse practitioner role definition and description, educational preparation, and quality and value of care are made and supported with authoritative, high-quality evidence.

As a nurse practitioner (NP) and NP educator, I am always looking for information on the nurse practitioner (NP) role in other countries. I was excited to see the recent review titled “Nurse Practitioner: Is It Time to Have a Role in Saudi Arabia? [1]” in *Nursing Reports*.

I appreciate that this is a new role to the authors, and I thank them for the information on the current practice climate for the NP role in Saudi Arabia. However, some inaccuracies in the definition and description of NPs should be clarified so that misconceptions are not perpetuated. The authors state that most NPs have doctoral degrees, and this is not the case. According to the AANP’s National Nurse Practitioner Sample Survey 2018, 17.8% of NPs report holding a doctoral degree [2]. The authors also state, “Having a Doctor of Nursing Practice [degree] is suggested for NPs to give them full independence in their practice.” These two things are not dependent on each other nor has it been suggested that they should be. Full practice authority is the preferred term instead of independent practice and a doctoral degree is not required for full practice authority. Full practice authority. “Full practice authority is generally defined as an NP’s ability to utilize knowledge, skills, and judgment to practice to the full extent of their education and training [3]. The American Association of Colleges of Nursing does, however, recommend that nurse practitioners obtain a doctoral-level education [4]. 

The section titled “The Value of the NP Role” is not necessarily inaccurate. However, I found it unfortunate that the highest quality evidence to support the value and quality of NP care was not cited. *Nursing Reports*’ readers deserve to know how strong the evidence is in support of the NP role. For over 20 years, studies have consistently provided evidence that NP care outcomes are similar if not superior to those of physicians. Some high-quality examples are Mundinger et al., 2000, a randomized trial published in JAMA on primary care outcomes in patients seen by NPs or physicians, the systematic review by Newhouse et al. in 2011 on the outcomes of patients treated by NPs, and, recently, the systematic review by Kleinpell et al. evaluating NP roles and management of patients in critical care [5,6,7]. 

I look forward to reading more about the progress of the NP role in Saudi Arabia and elsewhere in the world in the future.

## References

[1] Almutairi H.A., Alharbi K.N., Alotheimin H.K., Gassas R., Alghamdi M.S., Alamri A.A., Alsufyani A.M., Bashatah A.S. (2020). Nurse practitioner: Is it time to have a role in Saudi Arabia?. Nurs. Rep..

[2] American Association of Nurse Practitioners AANP National NP Sample Survey: Compensation and Practice 2019. https://www.aanp.org/practice/practice-related-research/research-reports.

[3] American Nurses Association (2020). ANA’s Principles for Advanced Practice Registered Nurse (APRN) Full Practice Authority. www.nursingworld.org/~49f695/globalassets/docs/ana/ethics/principles-aprnfullpracticeauthority.pdf.

[4] American Association of Colleges of Nursing AACN Position Statement on the Practice Doctorate in Nursing 2004. https://www.aacnnursing.org/Portals/42/News/Position-Statements/DNP.pdf.

[5] Kleinpell R., Grabenkort W., Robert P., Kapu A., Constantine R., Sicoutrism C. (2019). Nurse practitioners and physician assistants in acute and critical care: A concise review of the literature and data 2008–2018. Crit. Care Med..

[6] Mundinger M.O., Kane R.L., Lenz E.R., Totten A.M., Tsai W.Y., Cleary P.D., Friedewald W.T., Siu A.L., Shelanski M.L. (2000). Primary care outcomes in patients treated by nurse practitioners or physicians: A randomized trial. JAMA.

[7] Newhouse R.P., Stanik-Hutt J., White K.M., Johantgen M., Bass E.B., Zangaro G., Wilson R.F., Fountain L., Steinwachs D.M., Heindel L. (2011). Advanced practice nurse outcomes 1990–2008: A systematic review. Nurs. Econ..

